# Influence of inhaled nitric oxide on bronchopulmonary dysplasia in preterm infants with PPHN or HRF at birth: a propensity score matched study

**DOI:** 10.3389/fphar.2024.1515030

**Published:** 2024-12-11

**Authors:** Xue-rong Huang, Lian Wang, Guo-bao Liang, Sheng-qian Huang, Bao-ying Feng, Lu Zhu, Xu-fang Fan, Mu-lin Yao, Jing Zhang, Meng-jiao Wang, Zhi Zheng, Yao Zhu, Wen-li Duan, Zhan-kui Li, Jian Mao, Li Ma, Fa-lin Xu, Fan Wu, Qiu-fen Wei, Ling Liu, Xin-zhu Lin

**Affiliations:** ^1^ Department of Neonatology, Women and Children’s Hospital, School of Medicine, Xiamen University, Xiamen, Fujian, China; ^2^ Department of Pediatrics, Women and Children’s Hospital, School of Medicine, Xiamen University, Xiamen, Fujian, China; ^3^ Xiamen Key Laboratory of Perinatal-Neonatal Infection, Xiamen, China; ^4^ Department of Neonatology, Guiyang Maternal and Child Healthcare Hospital, Guiyang Children’s Hospital, Guiyang, Guizhou, China; ^5^ Department of Neonatology, Maternal and Child Health Hospital of the Guangxi Zhuang Autonomous Region, Nanning, Guangxi, China; ^6^ Department of Neonatology, The Third Affiliated Hospital of Guangzhou Medical University, Guangzhou, Guangdong, China; ^7^ Department of Neonatology, The Third Affiliated Hospital of Zhengzhou University, Zhengzhou, Henan, China; ^8^ Department of Neonatology, Children’s Hospital of Hebei province, Shijiazhuang, Hebei, China; ^9^ Department of Neonatology, Shengjing Hospital of China Medical University, Shenyang, Liaoning, China; ^10^ Department of Neonatology, Northwest Women’s and Children’s Hospital, Xi’an, Shanxi, China

**Keywords:** BPD (bronchopulmonary dysplasia), nitric oxide - NO, very premature infant, PPHN (persistent pulmonary hypertension of the newborn), hypoxemic respiratory failure

## Abstract

**Background:**

Bronchopulmonary Dysplasia (BPD) is a chronic lung disease affecting preterm infants, with limited prevention and treatment options. Inhaled Nitric Oxide (iNO) is sometimes used to treat Persistent Pulmonary Hypertension of the Newborn (PPHN) and Hypoxemic Respiratory Failure (HRF), and its impact on BPD development remains debated.

**Objective:**

To assess whether iNO-related factors are potential contributors to the development of BPD Grade Ⅱ-Ⅲ in very premature infants (VPI) diagnosed with PPHN or HRF at birth using Propensity Score Matching (PSM).

**Methods:**

We conducted a retrospective cohort study of infants born at 22–32 weeks gestation with PPHN or HRF, treated with iNO for over 3 h. PSM matched groups by gestational age, birth weight, and gender, etc. Multivariate logistic regression evaluated the association between iNO treatment and BPD outcomes to identify influencing factors, while Restricted Cubic Spline (RCS) and mediation analysis examined iNO dose effects and potential mediators like mechanical ventilation time and oxygenation index (OI).

**Results:**

A higher initial iNO dose was significantly associated with a reduced risk of BPD Grade Ⅱ-Ⅲ (*adjusted OR = 0.68, 95% CI: 0.52–0.89, p < 0.01*). Additionally, administration of iNO within the first 7 days of life was identified as an important influencing factor No significant mediation effects were observed for factors such as mechanical ventilation time and OI.

**Conclusion:**

A higher initial iNO dose within the first 7 days was associated with a reduced risk of BPD Grade Ⅱ-Ⅲ in VPI with PPHN or HRF.

## Background

BPD is a chronic lung disease that primarily affects VPI defined as infants born before 32 weeks of gestation. According to the 2019 Chinese Neonatal Collaborative Network, the overall incidence of BPD in VPIs admitted to 57 tertiary neonatal intensive care units (NICUs) was 29.2% (Cao et al., 2021). BPD results from an imbalance between lung injury and repair mechanisms, leading to long-term respiratory complications and neurodevelopmental issues (Sung, 2019)^,^ (Thekkeveedu et al., 2017). Its pathophysiological features include arrested alveolar development, reduced alveolar number, and impaired capillary growth (Dravet-Gounot et al., 2018). Key contributors to the development of BPD include inflammation, oxidative stress, and lung injury resulting from mechanical ventilation (Capasso et al., 2019). Current prevention and management of BPD require a comprehensive approach, including antenatal corticosteroids, surfactant therapy, protective lung ventilation strategies, and postnatal corticosteroids. However, these interventions have limited effectiveness and potential side effects (Gagliardi et al., 2007; Zozaya et al., 2019). Therefore, exploring new and more effective treatment methods is crucial.

NO, as an endogenous signaling molecule, has various important biological effects, including regulating pulmonary vascular tension during lung development (Nicholas and Channon, 2004). Early studies indicated that iNO could induce potent and selective pulmonary vasodilation, improving oxygenation (Liu et al., 2017). Several guidelines recommend iNO for treating term and late preterm infants with HRF and PPHN to reduce ECMO use. However, in recent years, off-label use of iNO in preterm infants (<34 weeks) has increased (Golombek and Young, 2010; Finer and Barrington, 2006; Cole et al., 2011). In VPI, studies on the use of iNO to reduce BPD have garnered significant attention. Preclinical data support iNO as a potential treatment for BPD prevention,It was found to improve oxygenation and reduces pulmonary resistance reduce pulmonary oxidative stress and inflammation, and promote alveolar development (Tourneux et al., 2009). in various animal experiments, Early use of iNO may help prevent BPD in preterm infants, reduce the need for respiratory support, and lower the risk of mechanical ventilation (Zheng et al., 2023).

Despite this, the efficacy of iNO in reducing BPD remains controversial. Hasan et al. (2017) conducted a randomized clinical trial involving 33 NICUs in the US and Canada, enrolling 451 preterm infants with a gestational age <30 weeks and birth weight <1250 g. They found that prolonged iNO treatment (24 days) did not reduce the risk of BPD. A multicenter trial in China also concluded that long-term, low-dose iNO treatment had no impact on the incidence of BPD in preterm infants (Jiang et al., 2016). The American Academy of Pediatrics (AAP) recommends that iNO should not be used for preventing BPD in preterm infants (Kumar et al., 2014).

VPI with PPHN or HRF are considered high-risk for BPD. Clinically, these infants sometimes receive NO treatment, yet some do not develop BPD or have milder forms of BPD. Whether this is related to NO-related factors has not been well-studied. The primary causes of HRF in very low birth weight (VLBW) infants were V/Q mismatch and pulmonary shunting due to surfactant deficiency. PPHN in this population had increasingly recognized as a cause of hypoxemia. INO has been shown to temporarily improve oxygenation in this group, as measured by partial pressure of oxygen (PaO2) and OI (Kinsella et al., 1999). This principle underlies the selective application of iNO based on physiological rationale for individual patients (Finer and Evans, 2015). NIH statements and the American Thoracic Society and Pediatric PH guidelines supported this individualized approach in specific subgroups of preterm infants (Cole et al., 2011; Abman et al., 2015; Kinsella et al., 2016). They recommend trying iNO treatment for preterm infants with severe hypoxemia secondary to PPHN physiology. Therefore, further precise investigation of NO-related factors in very preterm infants with PPHN or HRF is necessary to provide evidence for reducing BPD in preterm infants. This study mainly included VPI with PPHN or HRF at birth, using PSM to explore the influence of NO-related factors on the occurrence of BPD in these preterm infants.

## Materials and methods

### Study design

**FIGURE 1:**
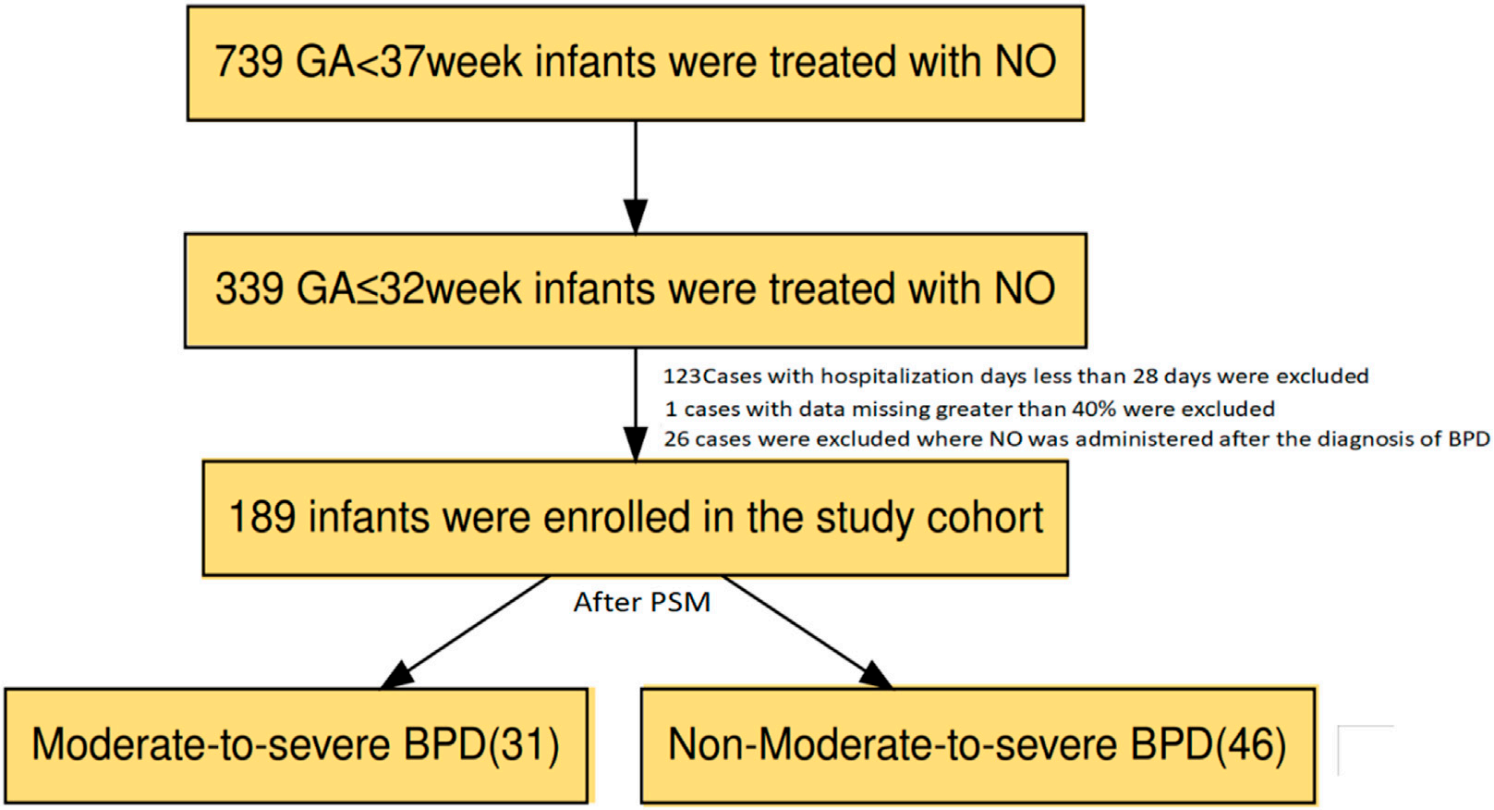
flow chart of the study.

The original study was a retrospective multicenter observational study conducted from January 2013 to December 2022. Data were collected from eight tertiary hospitals in five regions of China, all with NICU levels of III or higher (Hasan et al., 2017). Inclusion criteria: (1) gestational age (GA) 22–37 weeks; (2) receiving invasive respiratory support; (3) receiving iNO treatment for more than 3 h. Exclusion criteria: (1) congenital anomalies (including congenital diaphragmatic hernia and cyanotic congenital heart disease) or genetic metabolic diseases; (2) grade 3 or higher intracranial hemorrhage (IVH) before iNO treatment; (3) use of iNO for the prevention of BPD. The original study aimed to understand the use of iNO in preterm infants in mainland China. This study is a secondary utilization and in-depth analysis of the data collected in the cross-sectional survey. Inclusion criteria for this secondary study were: (1) GA of 22–32 weeks; (2) a primary diagnosis of HRF or PPHN confirmed at birth and treatment with inhaled nitric oxide (iNO).; Exclusion criteria for this secondary study were: (1) a hospital stay of ≤28 days; (2) missing data >40%.The flow chart is shown in Figure 1. The study subjects were divided into two group: BPD Grade Ⅱ-Ⅲand BPD Grade Ⅰ According to the 2018 NICHD standards, BPD was defined as the need for continuous oxygen therapy for 28 days or longer after birth or oxygen therapy at 36 weeks of corrected gestational age. BPD Grade Ⅱ-Ⅲ is defined as the need for respiratory support at 36 weeks of corrected gestational age. BPD Grade Ⅱ was defined as the need for low-flow oxygen (<2 L/min) or nasal continuous positive airway pressure (NCPAP) support; BPD Grade Ⅱwas defined as the need for high-flow oxygen (≥2 L/min) or mechanical ventilation support (Higgins et al., 2018).

#### Population enrollment

This study was registered in the Chinese Clinical Trial Registry (http://www.chictr.org.cn), registration number ChiCTR2200066935. The study protocol was approved by the Ethics Committee of Xiamen University Affiliated Women’s and Children’s Hospital/Xiamen Maternal and Child Health Hospital (KY-2023-019-H01). As this study was retrospective in nature, patient data were anonymized, and the requirement for individual informed consent was waived by the institutional ethics review board.

### Data collection

Data collection was conducted using a standardized survey form, including perinatal data of preterm infants and their mothers. This included: (1) Perinatal data of the preterm infants and their mothers; (2) Age at the time of iNO treatment, duration of iNO treatment, initial dose, and maximum dose; (3) Primary diseases of the preterm infants, postnatal complications, and survival outcomes.

### Definitions and diagnostic criteria of related diseases

(1) Effectiveness of iNO treatment: iNO treatment was considered effective if the FiO2 value decreased by ≥ 20% within 3 h of starting iNO treatment. If the FiO2 value remained unchanged, increased, or decreased by <20% after 3 h, iNO treatment was considered ineffective (Rallis et al., 2018); (2) HRF is defined as mechanical ventilation with an inhaled fraction of inspiration O2 (FiO2) ≥0.6, mean airway pressure (MAP)> 10 cmH2O, pre-ductal arterial partial pressure of oxygen (PaO2) <50 mmHg, percutaneous arterial oxygen saturation (SpO2) <85% or oxygenation index (OI)≥10 [OI = FiO2 ×MAP (cmH2O)× 100/PaO2 (mmHg) for >2 h without any ultrasound evidence of pulmonary Hypertension (PH) (Chandrasekharan et al., 2017). (3) Diagnosis criteria of PPHN: Clinical manifestations of hypoxemia, with echocardiographic evidence:Peak systolic pulmonary artery pressure (PAP) > 30 mmHg or >2/3 of systemic systolic pressure, estimated using the tricuspid regurgitation (TR) jet velocity via the modified Bernoulli equation; Functional markers, including right-to-left shunting at the ductal or atrial level and systolic septal flattening, were also incorporated, particularly in cases where TR velocity measurements were unavailable,If without echocardiography, a SpO2 difference of ≥5% between the right upper limb and right lower limb (Subspecialty Group of Neonatology et al., 2017; Clark et al., 2000); (3) Diagnosis criteria of early-onset sepsis (EOS): Refer to the expert consensus on the diagnosis and treatment of neonatal sepsis (2019 edition) (Subspecialty Group of Neonatology et al., 2019); (4) Hemodynamically significant patent ductus arteriosus (hsPDA): PDA diameter >1.5 mm, left atrium diameter/aortic diameter ≥1.5, accompanied by one of the following clinical manifestations: heart murmur, tachycardia (≥160 bpm), rapid breathing, widened pulse pressure (>25 mmHg), hypotension, bounding pulses, or cardiomegaly (Benitz et al., 2016); (5) Small for gestational age (SGA): Birth weight below the 10th percentile of the average birth weight for the same sex and gestational age (Sweet et al., 2023); (6) Diagnosis criteria of neonatal respiratory distress syndrome (nRDS): Based on the 2022 European RDS prevention and treatment guidelines (Papile et al., 1978); (7) Diagnosis and classification of intraventricular hemorrhage (IVH): Based on the Papile classification method (XM et al., 2017); (8) Diagnosis of sepsis, pulmonary hemorrhage, air leak syndrome, neonatal shock, etc.,: Refer to the “Practical Neonatology” (fifth edition) (Clark et al., 2010).

### Statistical analysis

Statistical analyses were performed using R language software (version 4.2.2, R Foundation for Statistical Computing, Vienna, Austria). Missing data were imputed using the random forest model (missForest package). Normally distributed quantitative data were described using mean ± standard deviation, and comparisons between groups were made using the independent sample *t*-test. Non-normally distributed quantitative data were described using median (interquartile range), and comparisons between groups were made using the Wilcoxon rank-sum test. Categorical variables were compared using the chi-square test. Differences with *p*-values less than 0.05 were considered statistically significant.

### PSM

To study whether inhalation of NO affects the occurrence of BPD Grade Ⅱ-Ⅲ, we used the PSM method, performing 1:2 nearest neighbor matching with a caliper of 0.1 to adjust for baseline characteristic differences between the two groups. Matching variables included gestational age, birth weight, sex, delivery mode, SGA, 1-min Apgar score, 5-min Apgar score, prenatal steroid use, prenatal steroid course, premature rupture of membranes (PROM) and duration, chorioamnionitis, maternal gestational diabetes mellitus (GDM), maternal preeclampsia, use of pulmonary surfactant and frequency, use of vasoactive drugs and duration, blood transfusion and amount, presence of PDA and PDA diameter, EOS, LOS, OI and P/F ratio before NO use.

After matching, standardized mean differences (SMD) were used to evaluate the balance of covariates between the two groups, with SMD less than 0.1 indicating ideal balance. Matching quality was displayed through balance diagnostic charts.

### Univariate and multivariate logistic regression analysis

Matched data were analyzed using univariate and conditional logistic regression to explore the impact of NO inhalation-related factors on the occurrence of BPD in preterm infants. Univariate analysis was used to identify potential risk factors (*p*-value threshold set at 0.05), followed by multivariate logistic regression. The significance level of the logistic regression model was set at α = 0.05, with differences considered statistically significant when bilateral *p* < 0.05.

### RCS analysis

We used RCS analysis to explore the relationship between the initial dose, maximum dose, and weight-standardized doses of iNO and the occurrence of BPD Grade Ⅱ-Ⅲ. RCS models can reveal the nonlinear relationship between iNO dose and the occurrence of BPD Grade Ⅱ-Ⅲ. We calculated the dose level inflection points and their corresponding OR (95% CI) and plotted dose-response curves to show the changes in the risk of BPD Grade Ⅱ-Ⅲ at different dose levels. The reference value of RCS depends on the shape of the RCS. In interpreting the results of RCS analysis, the choice of reference value for the predictor variable depends on the shape of the curve. If the curve is linear, the median of the predictor variable is chosen as the reference value. If the curve is U-shaped, inverted U-shaped, or L-shaped, the inflection point (i.e., the point where the curve changes direction) is set as the reference value. The inflection point represents the turning point or boundary between different patterns of the relationship between the predictor variable and the outcome.

### Mediation analysis

To evaluate the direct and indirect effects of inhaled NO dose on BPD Grade Ⅱ-Ⅲ, we conducted mediation analysis. Using OI and oxygen saturation (SpO2) before and after NO inhalation as mediating variables, we analyzed their mediation effects between NO dose and the occurrence of BPD. We used the mediation package to calculate total effect, direct effect, and indirect effect, and evaluated the 95% confidence interval of the mediation proportion through the Bootstrap method.

### Sensitivity analysis: repeated measures analysis of variance (ANOVA)

We used repeated measures ANOVA to evaluate the relationship between OI values at different time points and BPD Grade Ⅱ-Ⅲ. To address the correlation issue of repeated measures data, we conducted a sphericity test and adjusted the degrees of freedom using Greenhouse-Geisser and Huynh-Feldt methods when the sphericity assumption was not met. Homogeneity of variance was verified using Levene’s test to ensure the equality of variances between groups. Post hoc comparisons were used to identify significant differences between different time points, and estimated marginal means were used to show the trend over time.

Using the above statistical methods, we systematically analyzed the relationship between inhaled BPD Grade Ⅰ and BPD Grade Ⅱ-Ⅲ.

## Results

### Baseline characteristics and PSM results

In this study, we used the PSM method to analyze the risk factors for BPD in VPI who received iNO treatment. To ensure that the two groups were similar in terms of propensity scores and important covariates, we employed a 1:2 nearest neighbor matching method. The matched baseline characteristics included gestational age, birth weight, sex, delivery mode, SGA, 1-min Apgar score, 5-min Apgar score, prenatal steroid use, prenatal steroid course, PROM and duration, chorioamnionitis, maternal GDM, maternal preeclampsia, use of pulmonary surfactant and frequency, use of vasoactive drugs and duration, blood transfusion and amount, presence of PDA and PDA diameter, EOS, LOS, OI before iNO use, and P/F ratio. After matching, the analysis showed no significant differences in the main baseline characteristics between the two groups (SMD values less than 0.1) (see Supplementary Table S1).

### Post-matching basic analysis and conditional logistic regression analysis

After PSM, we further evaluated the differences in NO treatment and clinical outcomes between patients with BPD Grade Ⅰ and those with BPD Grade Ⅱ-Ⅲ. The results showed that the NO dose at the start of treatment was significantly lower in patients with BPD Grade Ⅱ-Ⅲ compared to those with BPD Grade Ⅰ (median: 8.0 ppm vs. 13.0 ppm, *p < 0.0012*). Additionally, the maximum NO dose received was also lower in the BPD Grade Ⅱ-Ⅲgroup (median: 10.0 ppm vs. 15.0 ppm, *p = 0.0402*). There were no significant differences between the two groups in terms of the duration of NO treatment and OI (see Table 1).

**TABLE 1:** Differences in NO treatment and Clinical outcomes after PSM.

Characteristic	BPD grade Ⅰ N = 46^a^	BPD grade Ⅱ-Ⅲ, N = 31^a^	*p*-value
Date of iNO Initiation (hours)	16 (7, 26)	14 (8, 45)	0.893^b^
Date of iNO Initiation≤168 h (7 days)	45 (97.8%)	25 (80.6%)	0.015^b^
Initial iNO Dose (ppm)	13.0 (8.3, 20.0)	8.0 (5.0, 10.0)	<0.001^b^
Maximum iNO Dose (ppm)	15.0 (8.3, 20.0)	10.0 (5.0, 15.0)	0.040^b^
Initial iNO Dose/BW	10.3 (5.4, 14.5)	5.7 (3.2, 10.1)	0.004^b^
Maximum iNO Dose/BW	11 (6, 15)	7 (4, 12)	0.059^b^
Duration of iNO Therapy (hours)	57 (38, 87)	56 (27, 111)	0.975^b^
Total Mechanical Ventilation	6 (4, 11)	7 (4, 22)	0.211^b^
Total High Frequency Ventilation Time	2.0 (1.0, 4.8)	5.0 (2.0, 7.0)	0.026^b^
Total Conventional Mechanical Ventilation	4 (1, 6)	2 (1, 12)	0.642^b^
Total Non-invasive Ventilation Time	19 (10, 28)	26 (11, 35)	0.113^b^
Total Oxygenation Time	38 (24, 58)	57 (43, 69)	0.002^b^
Platelet Count (10^9/L)	153 ± 75	149 ± 68	0.795^b^
Prothrombin Time (s)	17 (3, 19)	15 (13, 18)	0.783^b^
Fibrinogen Level (g/L)	1.90 (1.40, 2.16)	2.04 (1.63, 2.30)	0.149^b^
Activated Partial Thromboplastin Time	71 (66, 76)	68 (58, 79)	0.458^b^
Pre-treatment SpO2 (3 h prior)	7.8 (6.0, 10.9)	8.3 (6.6, 10.0)	0.747^b^
Post-treatment SpO2 (3 h after)	10 (4, 12)	10 (8, 12)	0.728^b^
SpO2 Difference (3 h)	−2.00 (−2.00, −1.00)	−2.00 (−2.00, −1.00)	0.269^b^
Oxygenation Index (3 h)	10 (9, 13)	10 (9, 12)	0.228^b^
P/F Ratio (3 h)	155 (102, 180)	154 (125, 179)	0.880^b^
Oxygenation Index (24 h)	7.7 (5.0, 11.2)	7.6 (4.7, 11.7)	0.839^b^
P/F Ratio (24 h)	178 (110, 219)	176 (128, 239)	0.751^b^
Oxygenation Index (48 h)	7.8 ± 4.2	8.4 ± 3.7	0.492^c^
P/F Ratio (48 h)	184 (143, 240)	164 (114, 213)	0.220^d^

Note:

^a^ Median (Interquartile Range); Mean ± Standard Deviation.

^b^ Wilcoxon.

^c^ *t*-test.

^d^ Wilcoxon rank sum exact test.

Regarding other clinical parameters, the median total mechanical ventilation time was slightly higher in patients with BPD Grade Ⅱ-Ⅲ, but the difference was not statistically significant (*p = 0.2112*). However, the total high-frequency ventilation time was significantly longer in patients with BPD Grade Ⅱ-Ⅲ(median: 5.0 vs. 2.0 h, *p* = 0.0262). Similarly, the total oxygen use time was significantly longer in patients withBPD Grade Ⅱ-Ⅲ(median: 57 vs. 38 h, *p = 0.0022*). Hematologic parameters such as platelet count and coagulation time showed no significant differences between the two groups, e. g., platelet count (*p = 0.7953*) and coagulation time (*p = 0.7832*).

Conditional logistic regression analysis further explored the relationship between NO dose and the severity of BPD. Univariate analysis indicated that an increase in the initial NO dose was significantly associated with a reduced risk of BPD Grade Ⅱ-Ⅲ (*adjusted OR = 0.86, 95% CI 0.79–0.95, p = 0.002*). Multivariate analysis supported this finding, showing that after controlling for potential confounders, an increase in the initial NO dose significantly reduced the risk of BPD Grade Ⅱ-Ⅲ (*adjusted OR = 0.68, 95% CI 0.51–0.90, p = 0.008*) (see Table 2. RCS Analysis (iNO Dose and BPD Grade Ⅱ-Ⅲ): RCS analysis showed a linear decrease in the risk of BPD Grade Ⅱ-Ⅲ with increasing initial iNO dose (*p* = 0.004), while the maximum iNO dose (inodose1) was not significantly associated with BPD risk (*p* = 0.065) (see Supplementary Figure S1).

**TABLE 2:** Conditional logistic regression analysis results after PSM.

Variables	Univariate Analysis					Multivariate Analysis				
	β	S.E	*Z*	*P*	OR (95%CI)	β	S.E	Z	*P*	OR (95%CI)
Date of iNO Initiation≤168 h (7 d)	−2.38	1.38	2.15	**0.032**	0.09 (0.01–0.81)	−3.16	1.45	2.18	**0.043**	0.06 (0.00–0.92)
Initial iNO Dose (ppm))	−0.15	0.05	−3.15	**0.002**	0.86 (0.79–0.95)	−0.39	0.15	−2.67	**0.008**	0.68 (0.51–0.90)
Duration of iNO Therapy (hours)	0.00	0.00	1.14	0.253	1.00 (1.00–1.01)	−0.00	0.00	−0.54	0.588	1.00 (0.99–1.00)
Maximum iNO Dose (ppm)	−0.08	0.04	−2.01	**0.044**	0.92 (0.85–0.99)	0.24	0.14	1.71	0.087	1.28 (0.96–1.69)

The bold values indicate statistically significant results (*p* < 0.05).

OR, Odds Ratio; CI, Confidence Interval.

### Mediation analysis between inhaled nitric oxide and BPD grade Ⅱ-Ⅲ

We conducted a mediation analysis to evaluate the relationship between the initial and maximum doses of iNO and BPD Grade Ⅱ-Ⅲ, including weight-standardized doses. The analysis results (see Supplementary Tables S2–S5) showed that at the initial dose, the post-ductal SpO2 and OI 3 h after iNO inhalation exhibited certain mediation effects, with mediation proportions of 2.7% (*95% CI - 17.4, 18.8*) and 3.0% (*95% CI - 7.7, 23.4*), respectively. The pre-ductal SpO2 3 h after inhalation, OI at 24 h, and OI at 48 h did not show significant mediation effects, with mediation proportions close to 0%, −3.2%, and −0.9%, respectively. For the maximum dose and its weight-standardized values, none of the time points for SpO2 and OI showed significant mediation effects. These results indicate that while the dose of iNO directly reduced the risk of BPD Grade Ⅱ-Ⅲ, its indirect effects through changes in SpO2 and OI were not significant.

### Relationship between OI values at different time points and BPD grade Ⅱ-Ⅲ (Repeated measures ANOVA)

In this study, we used repeated measures ANOVA to evaluate the relationship between OI values at different time points and BPD Grade Ⅱ-Ⅲ.

Sphericity and Homogeneity of Variance Tests (see Supplementary Tables S6, S7): The sphericity test failed (*Mauchly’s W = 0.4262142, p < 0.001*), indicating that the covariance matrix of the repeated measures did not meet the sphericity assumption. Therefore, we adjusted the degrees of freedom using Greenhouse-Geisser (*ε = 0.6617726*) and Huynh-Feldt (*ε = 0.6796996*) methods. The homogeneity of variance test results showed that the *p*-values at all time points were greater than 0.05, indicating that the variances between different groups were equal, meeting the homogeneity of variance assumption.

Repeated Measures ANOVA Results (see Table 3): Within-subjects effects (time effects): Time had a significant impact on OI values (*F = 47.121, p < 0.001, η* ^*2*^ *G = 0.296*). This indicates significant differences in OI values at different time points (oi0, oi24, oi48), with approximately 29.65% of the variance explained by time.

**TABLE 3:** Within subjects effects.

	Correction	Sum of squares	df	Mean square	F	*p*-value	η^2^G
Times	Greenhouse-Geisser	6,546.65542	1.985318	3,297.53525	47.1210014	<0.001	0.296510781
Times:BPD Grade Ⅱ-Ⅲ	Greenhouse-Geisser	57.69261	1.985318	29.05963	0.4152553	0.659	0.003700612
Residual	Greenhouse-Geisser	10,419.96439	148.898835	69.98016			

Note. Type 3 Sums of Squares.

Between-subjects effects (impact of BPD Grade Ⅱ-Ⅲ) (see Table 4): There was no significant between-subjects effect between BPD Grade Ⅱ-Ⅲ and OI values (*F = 0.060, p = 0.808, η* ^*2*^ *G = 0.00026)*, indicating no differences in OI values between groups BPD Grade Ⅰ and BPD Grade Ⅱ-Ⅲ.

**TABLE 4:** Between subjects effects.

	Sum of squares	df	Mean square	F	*p*-value	η^2^G
BPD Grade Ⅱ-Ⅲ	4.057837	1	4.057837	0.0595298	0.808	0.0002611829
Residual	5,112.359559	75	68.164794			

Note. Type 3 Sums of Squares.

Post hoc comparisons (see Table 5): The *post hoc* comparison results showed a significant decrease in OI values from oi0 to subsequent time points (oi24, oi48), especially from oi0 to oi48 (*p < 0.001*). This indicates a significant improvement in OI values over time. The change from oi0 to oi48 was also significant (*p < 0.001*), while the change from oi24 to oi48 was not significant.

**TABLE 5:** Post hoc comparisons—times.

Times1	Times2	Mean difference	SE	df	t	P (Tukey)
oi0	oi24	10.696483	1.4570476	75	7.341203	<0.001
oi0	oi48	11.990900	1.2801314	75	9.366929	<0.001
oi24	oi48	1.294417	0.7888473	75	1.640897	0.362

Adjusted by BPD Grade Ⅱ-Ⅲ.

Note: Oi0, Worst oxygenation index before iNO; treatment; Oi24, Oxygenation Index at 24 h; Oi48, Oxygenation Index at 48 h.

Estimated Marginal Means (*see* Table 6): The OI values at each time point showed changes over time, regardless of the presence of BPD. Although these changes did not significantly differ between BPD groups, overall, the OI values significantly changed over time.

**TABLE 6:** Estimated marginal means for OI by times: BPD grade Ⅱ-Ⅲ.

BPD grade Ⅱ-Ⅲ	Times	Mean	SE	95% CI lower	95% CI upper
0	oi0	19.511304	1.5587661	16.406083	22.616526
0	oi24	9.128330	1.0754303	6.985963	11.270696
0	oi48	7.755694	0.5853806	6.589556	8.921832
1	oi0	20.603000	1.8987991	16.820398	24.385602
1	oi24	9.593009	1.3100272	6.983301	12.202717
1	oi48	8.376811	0.7130769	6.956289	9.797333

Adjusted by BPD Grade Ⅱ-Ⅲ.

Note: Oi0, Worst oxygenation index before iNO; treatment; Oi24, Oxygenation Index at 24 h; Oi48, Oxygenation Index at 48 h.

The results of this study indicate that time has a significant impact on OI values, showing significant improvement over time. However, the changes in OI values did not significantly differ between groups BPD Grade Ⅰ and BPD Grade Ⅱ-Ⅲ.

## Discussion

The use of iNO in preterm infants remains controversial among clinicians. In 2014, the American Academy of Pediatrics Committee on Fetus and Newborn issued a statement that current data do not support the routine use of iNO in preterm infants. Despite the lack of significant benefits, data from California in 2016 showed an increase in the use of iNO in preterm infants (Clark et al., 2010). A systematic review in 2017 indicated that iNO did not provide significant advantages in preterm infants. However, this review noted that populations such as preterm infants with pulmonary hypertension had not been individually studied (Cole et al., 2011). Additionally, recent non-randomized controlled trials have suggested that iNO may benefit specific subgroups of preterm infants, particularly those with PPHN, prolonged rupture of membranes, and prenatal steroid exposure. These studies indicate that under specific clinical and experimental conditions, iNO may have potential benefits for lung development and function in preterm infants (Vieira et al., 2021). Therefore, this study selected VPI with early PPHN or HRF for iNO treatment, using the PSM method to eliminate confounding factors. The results showed significant differences in iNO-related factors between the BPD Grade Ⅰand BPD Grade Ⅱ-Ⅲ. Subsequent multivariate regression analysis indicated that the initial iNO dose significantly reduced the risk of BPD Grade Ⅱ-Ⅲ(adjusted OR = 0.68, 95% CI 0.51-0.90, *p* = 0.008), while the increase in maximum NO dose did not reach statistical significance in the multivariate analysis. RCS analysis showed a linear decrease in the risk of BPD Grade II-III with increasing initial iNO dose (*p* = 0.004). This study supports the correlation between iNO-related factors and the occurrence of BPD, further emphasizing the potential benefits of iNO treatment in specific subgroups of preterm infants. The mechanisms by which iNO may reduce BPD risk extend beyond its direct effects on pulmonary function. Potential pathways include the reduction of pulmonary inflammation, improvement in vascular remodeling, and modulation of immune responses, which may collectively support lung development and mitigate injury. Some studies suggested that if pulmonary vascular dysfunction or ventilation-perfusion mismatch is not part of the specific patient’s pathophysiology, treatments targeting this pathway are unlikely to be beneficial (Sherlock et al., 2020). Therefore, iNO treatment should be individualized based on clear pathophysiological foundations to maximize clinical benefits.

Literature indicates that the timing of initial iNO treatment significantly affects its effectiveness in reducing the risk of BPD. Studies have found that starting iNO treatment early (≤3 days) in preterm infants with PPHN significantly improves treatment efficacy and survival rates (Rallis et al., 2018). A meta-analysis showed that starting iNO treatment within 7 days after birth reduces the risk of BPD, while starting treatment after 7 days does not show significant statistical differences (Rallis et al., 2018). These studies highlight the importance of early iNO treatment, particularly within 3 days after birth, which may bring significant clinical benefits. In this study, after PSM, our data showed that the median start time for NO treatment was 16 h (IQR: 7 h, 26 h) in the BPD Grade Ⅰ group and 14 h (IQR: 8h, 45 h) in the BPD Grade Ⅱ-Ⅲgroup, with no significant differences between the two groups (*p* = 0.8932). However, the proportion of NO treatment started within 7 days after birth was significantly higher in the BPD Grade Ⅰ group compared to the BPD Grade Ⅱ-Ⅲgroup (97.8% vs. 80.6%, *p* = 0.0152). This is consistent with the evidence from the aforementioned literature (Wang et al., 2021), further supporting the importance of early iNO treatment, suggesting that early treatment may be crucial in reducing the severity of BPD. Early iNO treatment may improve oxygenation and pulmonary vascular function in preterm infants, reduce pulmonary inflammation and oxidative stress, thereby lowering the risk of BPD. This mechanism is supported by both preclinical and clinical studies (Tourneux et al., 2009)^.^ Early (within 7 days after birth) administration of iNO may be a key strategy in reducing the severity of BPD in preterm infants.

Studies indicate that the dosage of iNO treatment significantly impacts its effectiveness in reducing the risk of BPD. Dosages ranging from 5 to 20 ppm are considered effective in controlling PPHN and HRF in neonates (Kumar et al., 2014). Recent meta-analyses indicated that iNO was effective at doses higher than 5 ppm, with an initial dose of 10 ppm showing effectiveness in reducing the risk of BPD in preterm infants (Hasan et al., 2017). In this study, the NO dosage ranged from 2 to 20 ppm, not exceeding the guideline-recommended 20 ppm. Within this range, our study found that at the guideline-recommended 5–20 ppm, higher initial doses were associated with a significant reduction in the severity of BPD. After PSM, univariate results showed that the initial iNO dose at the start of treatment was significantly lower in the BPD Grade Ⅱ-Ⅲgroup compared to the BPD Grade Ⅰ group (8.0 ppm vs. 13.0 ppm, *p* < 0.0012), and the maximum iNO dose was also lower (10.0 ppm vs. 15.0 ppm, *p* = 0.0402). There were no significant differences between the two groups in terms of the duration of iNO treatment and OI. Conditional logistic regression analysis showed that each unit increase in initial iNO dose was significantly associated with a reduced risk of BPD Grade Ⅱ-Ⅲ(adjusted *OR = 0.86, 95% CI 0.79–0.95, p = 0.002*), and each unit increase in maximum iNO dose was also significantly associated (adjusted *OR = 0.92, 95% CI 0.85–0.99, p = 0.044*). In multivariate analysis, an increase in initial iNO dose significantly reduced the risk of BPD Grade Ⅱ-Ⅲ(adjusted *OR = 0.68, 95% CI 0.51–0.90, p = 0.008*), while the increase in maximum iNO dose did not reach statistical significance. RCS analysis further revealed a linear association between the initial iNO dose and the risk of BPD Grade Ⅱ-Ⅲ(*OR = 0.48, 95% CI 0.27–0.82, p = 0.010*). Combining literature and the results of this study, we found that higher initial NO doses are significantly associated with the reduction in the severity of BPD. Guidelines suggested that due to the reduced sensitivity to high concentrations of NO after the ineffectiveness of low-concentration NO inhalation, the initial treatment concentration should be 20 ppm (Hong et al., 2019). Two large multicenter RCT studies recommended to start at 20 ppm, reduce NO by 5 ppm every 4 h, reduce NO demand after 24 h of treatment, and stop NO treatment after 96 h (Haseoet et al., 2005)^,^ (Clark et al., 2010). A meta-analysis suggests that an initial NO treatment concentration of 20 ppm is effective for HRF in term or late preterm infants (Zheng et al., 2023). In summary, the results of this study are consistent with findings in the literature, emphasizing the importance of initial treatment dose and early treatment in reducing the risk of BPD. In clinical practice, considering the specific conditions of individual patients and appropriately adjusting the initial dose and treatment strategy of iNO may be key factors in reducing the occurrence of BPD in preterm infants.

In this study, we further explored the role of mechanical ventilation time and OI in reducing the severity of BPD through iNO treatment using mediation analysis and ANOVA. Mediation analysis showed that although initial and maximum iNO doses significantly impacted the risk of BPD Grade Ⅱ-Ⅲ, these effects were not mediated by mechanical ventilation time and OI. Specifically, the mediation effects between initial iNO dose and mechanical ventilation time and OI did not reach statistical significance. This suggests that while mechanical ventilation time and oxygenation status are important factors affecting the occurrence of BPD (Naeem et al., 2019), these factors did not exhibit significant mediation effects during iNO treatment in this study. In other words, the main mechanism by which iNO reduces the risk of BPD may not rely on these mediation factors. Repeated measures ANOVA results further supported this conclusion. We evaluated the relationship between OI values at different time points and the occurrence of BPD Grade Ⅱ-Ⅲ. The results showed significant differences in OI values at different time points (oi0, oi24, oi48), with significant improvement over time. However, this change did not significantly differ between groups BPD Grade Ⅰ and BPD Grade Ⅱ-Ⅲ, indicating that while the improvement in OI values was significant, it did not significantly affect the severity of BPD. Existing literature indicates that mechanical ventilation time and oxygenation status directly impact the occurrence of BPD. Early studies suggest that iNO treatment in preterm infants significantly improves oxygenation status, reduces pulmonary inflammation and oxidative stress, thereby lowering the risk of BPD (Ellsworth et al., 2015). However, the results of mediation analysis and repeated measures ANOVA in this study indicate that mechanical ventilation time and OI are not the main mediation factors by which iNO reduces the risk of BPD. This finding may suggest that iNO directly improves pulmonary vascular function in preterm infants through other mechanisms, reducing the accumulation of lung injury and thus lowering the risk of BPD. Combining literature and the findings of this study, we speculate that early intervention and optimization of initial iNO dose are key factors in reducing the risk of BPD, and these effects do not entirely depend on changes in mechanical ventilation time and oxygenation status.

Although this study explored the impact of iNO treatment on BPD risk in preterm infants using PSM and various statistical methods, several limitations exist. First, the sample size is limited, and despite PSM reducing the impact of confounding factors, some subgroup analyses may lack statistical power. Although PSM reduces some biases, unmeasured confounding factors, such as baseline health status, nutritional support, and adjunctive treatments, may still affect outcomes. Additionally, this retrospective study is subject to selection and information biases. Another major limitation is the high mortality rate (36%) in the iNO-treated cohort, which may bias the results and affect the reliability of conclusions regarding iNO’s impact on BPD. Notable limitation involves the diagnosis of PPHN. The reliance on PAP measurements, particularly in preterm infants, is challenging due to the difficulty in capturing TR signals. The cohort was exclusively drawn from tertiary hospitals in China, limiting generalizability. Regional differences in neonatal care practices may influence outcomes, underscoring the need for multicenter studies in diverse settings to improve applicability. Future research should validate and expand these findings through prospective randomized controlled trials, larger sample sizes, and multicenter studies to provide more reliable evidence for reducing the severity of BPD in preterm infants.

## Conclusion

This study shows that A higher initial iNO dose within the first 7 days was associated with a reduced risk of BPD Grade Ⅱ-Ⅲ in VPI with PPHN or HRF, independent of changes in mechanical ventilation time and oxygenation status. The findings support current guidelines recommended an initial dose of 20 ppm, emphasizing the importance of early and appropriately dosed treatment. Future research should explore additional mediators to optimize treatment strategies and further reduce BPD incidence.

## Data Availability

The original contributions presented in the study are included in the article/Supplementary Material, further inquiries can be directed to the corresponding authors.
