# Supplementary material for: Influence of inhaled nitric oxide on bronchopulmonary dysplasia in preterm infants with PPHN or HRF at birth: a propensity score matched study
Source: Front Pharmacol. 2024 Dec 11;15:1515030. doi: 10.3389/fphar.2024.1515030 (PMC11670073; doi:10.3389/fphar.2024.1515030)
Supplement: Supplementary file 1 [file DataSheet1.docx]

**Supplementary Information**

**TableS1: Baseline Data Before and After PSM Matching**

| **Variables** | **Before PSM** | | | | **after PSM** | | | |
| --- | --- | --- | --- | --- | --- | --- | --- | --- |
|  | Total (n = 189) | NO (n = 117) | YES (n = 72) | SMD | Total (n = 77) | YES (n = 31) | NO (n = 46) | SMD |
| Gestational Age M (Q₁ Q₃) | 29.00 (28.00, 31.00) | 31.00 (29.00, 32.00) | 28.50 (27.00, 29.25) | -0.984 | 29.00 (28.00, 30.00) | 29.00 (28.00, 31.00) | 29.00 (28.00, 30.00) | -0.261 |
| Days of Gestation M (Q₁ Q₃) | 2.00 (1.00, 5.00) | 2.00 (1.00, 5.00) | 2.00 (1.00, 4.00) | -0.027 | 2.00 (1.00, 5.00) | 3.00 (1.00, 5.00) | 2.00 (1.00, 3.00) | -0.548 |
| Birth Weight M (Q₁ Q₃) | 1390.00 (1120.00, 1630.00) | 1500.00 (1260.00, 1700.00) | 1200.00 (887.50, 1400.00) | -0.790 | 1360.00 (1090.00, 1500.00) | 1375.00 (1125.00, 1498.75) | 1310.00 (1085.00, 1527.50) | -0.225 |
| Birth Weight Fenton Percentile M (Q₁ Q₃) | 0.55 (0.38, 0.72) | 0.55 (0.37, 0.69) | 0.56 (0.41, 0.78) | 0.098 | 0.57 (0.43, 0.76) | 0.53 (0.37, 0.76) | 0.59 (0.51, 0.76) | 0.445 |
| Apgar Score at 1 minute M (Q₁ Q₃) | 8.00 (6.00, 9.00) | 8.00 (7.00, 9.00) | 7.00 (5.75, 8.00) | -0.375 | 8.00 (6.00, 9.00) | 8.00 (6.00, 9.00) | 7.00 (5.50, 8.00) | -0.344 |
| Apgar Score at 5 minutes M (Q₁ Q₃) | 9.00 (8.00, 9.00) | 9.00 (8.00, 9.00) | 8.11 (8.00, 9.00) | -0.160 | 9.00 (8.00, 9.00) | 9.00 (8.00, 9.00) | 8.00 (8.00, 9.00) | -0.240 |
| Hormone Treatment Duration M (Q₁ Q₃) | 1.00 (0.00, 1.00) | 1.00 (0.00, 1.00) | 1.00 (0.00, 1.00) | 0.060 | 1.00 (0.88, 1.00) | 1.00 (0.98, 1.00) | 1.00 (0.84, 1.00) | -0.063 |
| PROM Duration M (Q₁ Q₃) | 0.00 (0.00, 48.00) | 0.00 (0.00, 84.00) | 0.00 (0.00, 15.75) | -0.349 | 0.00 (0.00, 47.00) | 0.00 (0.00, 96.00) | 0.00 (0.00, 10.00) | -0.230 |
| Surfactant Usage Times M (Q₁ Q₃) | 2.00 (1.00, 2.00) | 2.00 (1.00, 2.00) | 2.00 (1.00, 2.00) | 0.193 | 2.00 (1.00, 3.00) | 2.00 (1.00, 3.00) | 2.00 (1.00, 3.00) | -0.032 |
| Vasoactive Drug Usage (Days) M (Q₁ Q₃) | 12.00 (1.00, 123.00) | 12.00 (1.00, 26.00) | 12.00 (1.00, 123.00) | 0.161 | 12.00 (1.00, 16.00) | 12.00 (1.25, 123.00) | 12.00 (0.50, 12.00) | -4.351 |
| Number of Vasoactive Drugs Used M (Q₁ Q₃) | 5.00 (1.00, 11.00) | 4.00 (1.00, 9.00) | 6.00 (2.75, 12.25) | 0.164 | 6.00 (1.00, 10.00) | 6.00 (1.00, 10.75) | 5.00 (1.00, 9.50) | -0.370 |
| Lowest pH Value before iNO M (Q₁ Q₃) | 7.24 (7.17, 7.30) | 7.24 (7.15, 7.29) | 7.26 (7.20, 7.32) | 0.240 | 7.26 (7.21, 7.31) | 7.26 (7.21, 7.31) | 7.25 (7.20, 7.32) | -0.160 |
| Worst OI before iNO M (Q₁ Q₃) | 19.00 (11.00, 26.99) | 16.70 (9.90, 25.11) | 21.83 (14.38, 28.25) | 0.308 | 20.00 (11.90, 25.60) | 20.93 (11.83, 25.48) | 19.20 (12.75, 26.05) | 0.085 |
| Worst P/F Ratio before iNO M (Q₁ Q₃) | 67.86 (49.25, 109.00) | 70.26 (50.00, 121.60) | 61.70 (47.37, 85.75) | -0.350 | 61.40 (47.60, 91.67) | 60.43 (47.15, 88.58) | 68.60 (49.80, 94.78) | 0.007 |
| Number of Transfusions M (Q₁ Q₃) | 2.47 (2.00, 5.00) | 2.17 (1.98, 4.00) | 3.29 (2.00, 6.00) | 0.454 | 2.45 (2.00, 4.00) | 2.48 (2.00, 4.00) | 2.11 (1.79, 4.00) | 0.027 |
| Total RBC Transfused M (Q₁ Q₃) | 73.31 (47.00, 126.33) | 55.76 (40.00, 101.19) | 104.21 (73.47, 183.20) | 0.561 | 69.27 (43.65, 108.00) | 59.92 (42.50, 116.50) | 78.01 (45.19, 104.16) | -0.099 |
| Malen (%) | 128 (67.72) | 79 (67.52) | 49 (68.06) | 0.011 | 52 (67.53) | 31 (67.39) | 21 (67.74) | 0.008 |
| Delivery Method n (%) |  |  |  |  |  |  |  |  |
| Vaginal Delivery | 75 (39.68) | 42 (35.90) | 33 (45.83) | 0.199 | 23 (29.87) | 12 (26.09) | 11 (35.48) | 0.196 |
| Cesarean Section | 114 (60.32) | 75 (64.10) | 39 (54.17) | -0.199 | 54 (70.13) | 34 (73.91) | 20 (64.52) | -0.196 |
| Place of Birth （In-Hospital）n (%) | 155 (82.01) | 94 (80.34) | 61 (84.72) | 0.122 | 69 (89.61) | 41 (89.13) | 28 (90.32) | 0.040 |
| Antenatal Steroids Usage n (%) |  |  |  |  |  |  |  |  |
| No Usage | 53 (28.04) | 32 (27.35) | 21 (29.17) | 0.040 | 11 (14.29) | 6 (13.04) | 5 (16.13) | 0.084 |
| Incomplete Course | 54 (28.57) | 39 (33.33) | 15 (20.83) | -0.308 | 26 (33.77) | 15 (32.61) | 11 (35.48) | 0.060 |
| Complete Course | 82 (43.39) | 46 (39.32) | 36 (50.00) | 0.214 | 40 (51.95) | 25 (54.35) | 15 (48.39) | -0.119 |
|  | 25 (13.23) | 14 (11.97) | 11 (15.28) | 0.092 | 9 (11.69) | 5 (10.87) | 4 (12.90) | 0.061 |
| IVF n (%) | 48 (25.4) | 26 (22.22) | 22 (30.56) | 0.181 | 23 (29.87) | 14 (30.43) | 9 (29.03) | -0.031 |
| Gestational Diabetes n (%) | 38 (20.11) | 25 (21.37) | 13 (18.06) | -0.086 | 11 (14.29) | 7 (15.22) | 4 (12.90) | -0.069 |
| Hypertension n (%) | 24 (12.7) | 15 (12.82) | 9 (12.50) | -0.010 | 8 (10.39) | 7 (15.22) | 1 (3.23) | -0.679 |
| Chorioamnionitis n (%) | 25 (13.23) | 18 (15.38) | 7 (9.72) | -0.191 | 8 (10.39) | 6 (13.04) | 2 (6.45) | -0.268 |
| Multiple Pregnancy n (%) | 59 (31.22) | 37 (31.62) | 22 (30.56) | -0.023 | 26 (33.77) | 16 (34.78) | 10 (32.26) | -0.054 |
| Triplets or More | 4 (2.12) | 1 (0.85) | 3 (4.17) | 0.166 | 3 (3.9) | 1 (2.17) | 2 (6.45) | 0.174 |
| PROM n (%) | 71 (37.57) | 47 (40.17) | 24 (33.33) | -0.145 | 30 (38.96) | 20 (43.48) | 10 (32.26) | -0.240 |
| Amniotic Fluid Contamination（3stage）, n (%) | 5 (2.65) | 3 (2.56) | 2 (2.78) | 0.013 | 1 (1.3) | 1 (2.17) | 0 (0.00) | -0.193 |
| Placental Abruption, n (%) | 24 (12.7) | 16 (13.68) | 8 (11.11) | -0.082 | 10 (12.99) | 9 (19.57) | 1 (3.23) | -0.925 |
| PS Usage, n (%) | 178 (94.18) | 111 (94.87) | 67 (93.06) | -0.071 | 74 (96.1) | 44 (95.65) | 30 (96.77) | 0.064 |
| Blood Transfusion, n (%) | 153 (80.95) | 88 (75.21) | 65 (90.28) | 0.508 | 60 (77.92) | 35 (76.09) | 25 (80.65) | 0.115 |
| HsPDA Ratio, n (%) | 55(29.10) | 30 (25.6) | 22 (30.6) | 0.463 | 20(26.59) | 9 (29.01) | 11 (23.91) | 0.615 |
| PDA Diameter Ratio, n (%) |  |  |  |  |  |  |  |  |
| ≥0.15and≤0.24cm | 49 (25.93) | 25 (21.37) | 24 (33.33) | 0.254 | 21 (27.27) | 14 (30.43) | 7 (22.58) | -0.188 |
| ≥0.24cm | 65 (34.39) | 46 (39.32) | 19 (26.39) | -0.293 | 28 (36.36) | 17 (36.96) | 11 (35.48) | -0.031 |
| Eos, n (%) | 39 (20.63) | 25 (21.37) | 14 (19.44) | -0.049 | 18 (23.38) | 13 (28.26) | 5 (16.13) | -0.330 |
| Los, n (%) | 12 (6.35) | 4 (3.42) | 8 (11.11) | 0.245 | 4 (5.19) | 2 (4.35) | 2 (6.45) | 0.086 |
| Postnatal Steroids, n (%) | 59 (31.22) | 21 (17.95) | 38 (52.78) | 0.698 | 27 (35.06) | 16 (34.78) | 11 (35.48) | 0.015 |
| IUGR, n (%) | 6 (3.17) | 2 (1.71) | 4 (5.56) | 0.168 | 1 (1.3) | 1 (2.17) | 0 (0.00) | -0.193 |

***Note:****PROM: Premature Rupture of Membranes、iNO: Inhaled Nitric Oxide、OI: Oxygenation Index、P/F Ratio: PaO₂/FiO₂ Ratio、RBC: Red Blood Cells、IVF: In Vitro Fertilization、PDA: Patent Ductus Arteriosus、PS: Pulmonary Surfactant、Eos: Early Onset Sepsis、Los: Late Onset Sepsis、IUGR: Intrauterine Growth Restriction*


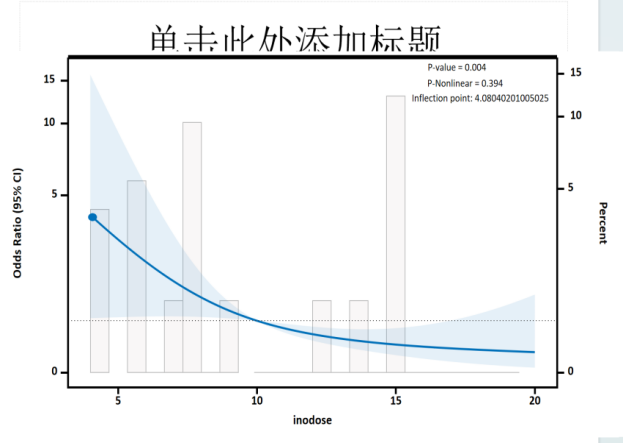

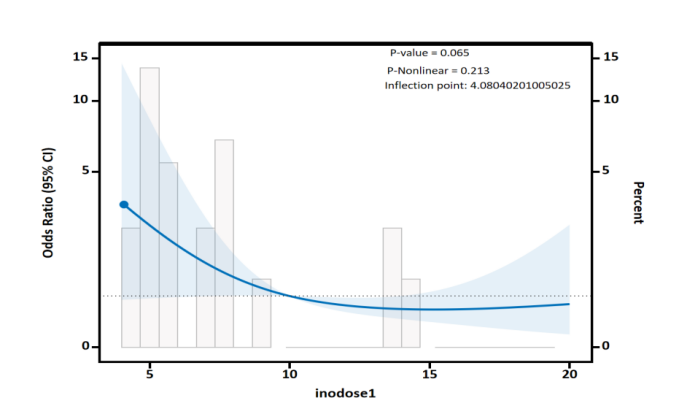


A B

**Figure S1: Association between iNO Dose and BPD Using the RCS Function**

(A) Initial iNO dose and BPD. (B) Maximum iNO dose and BPD. Each model uses Restricted Cubic Spline (RCS) functions with knots located at the 10th, 50th, and 90th percentiles,. The Y-axis represents the odds ratio (OR) for developing BPD compared to individuals with the reference value (50th percentile) of the respective dose metric.

**Table S2: Mediation analysis for the associations between Initial iNO dose and Moderate-to-Severe BPD**

| **Independent variable** | **Mediator** | **Total effect** | | **Indirect effect** | | **Direct effect** | | **Proportion mediated, % (95% CI)** |
| --- | --- | --- | --- | --- | --- | --- | --- | --- |
|  |  | **Coefficient (95% CI)** | **P value** | **Coefficient (95% CI)** | **P value** | **Coefficient (95% CI)** | **P value** |  |
| Initial iNO dose | SpO2_pre3 | -0.02793 (-0.03663, -0.01616) | <0.001 | -0.00001 (-0.00292, 0.00182) | 0.772 | -0.02791 (-0.03652, -0.01567) | <0.001 | 0.0 (-7.5, 11.2) |
| Initial iNO dose | **SpO2_post3** | -0.02805 (-0.03689, -0.01623) | <0.001 | -0.00077 (-0.00508, 0.00470) | 0.664 | -0.02728 (-0.03784, -0.01434) | <0.001 | 2.7 (-17.4, 18.8) |
| Initial iNO dose | oi3 | -0.02801 (-0.03635, -0.01658) | <0.001 | -0.00085 (-0.00586, 0.00217) | 0.576 | -0.02716 (-0.03691, -0.01393) | <0.001 | 3.0 (-7.7, 23.4) |
| Initial iNO dose | oi24 | -0.02784 (-0.03628, -0.01577) | <0.001 | 0.00090 (-0.00238, 0.00421) | 0.564 | -0.02874 (-0.03835, -0.01609) | <0.001 | -3.2 (-16.9, 9.1) |
| Initial iNO dose | oi48 | -0.02775 (-0.03605, -0.01597) | <0.001 | 0.00026 (-0.00216, 0.00359) | 0.876 | -0.02801 (-0.03726, -0.01591) | <0.001 | -0.9 (-12.2, 9.1) |

Note: SpO2 pre3: SpO2 change 3 hours after NO use (pre-ductal); SpO2 post3: SpO2 change 3 hours after NO use (post-ductal); Oi3: Oxygenation Index at 3 hours; Oi24: Oxygenation Index at 24 hours; Oi48: Oxygenation Index at 48 hours

**TableS3: Mediation analysis for the associations between Maximum iNO dose and Moderate-to-Severe BPD**

| **Independent variable** | **Mediator** | **Total effect** | | **Indirect effect** | | **Direct effect** | | **Proportion mediated, % (95% CI)** |
| --- | --- | --- | --- | --- | --- | --- | --- | --- |
|  |  | **Coefficient (95% CI)** | **P value** | **Coefficient (95% CI)** | **P value** | **Coefficient (95% CI)** | **P value** |  |
| Maximum iNO dose | SpO2_pre3 | -0.01860 (-0.02980, -0.00182) | 0.032 | -0.00002 (-0.00338, 0.00215) | 0.876 | -0.01858 (-0.02985, -0.00169) | 0.032 | 0.1 (-12.2, 31.7) |
| Maximum iNO dose | **SpO2_post3** | -0.01877 (-0.02993, -0.00209) | 0.028 | -0.00098 (-0.00525, 0.00306) | 0.576 | -0.01780 (-0.02949, -0.00115) | 0.044 | 5.2 (-28.0, 46.4) |
| Maximum iNO dose | oi3 | -0.01859 (-0.02986, -0.00197) | 0.028 | -0.00137 (-0.00750, 0.00404) | 0.632 | -0.01721 (-0.02938, 0.00071) | 0.064 | 7.4 (-48.2, 85.0) |
| Maximum iNO dose | oi24 | -0.01854 (-0.02949, -0.00174) | 0.032 | 0.00055 (-0.00296, 0.00315) | 0.656 | -0.01909 (-0.03053, -0.00211) | 0.028 | -3.0 (-37.5, 18.9) |
| Maximum iNO dose | oi48 | -0.01856 (-0.02995, -0.00179) | 0.028 | 0.00103 (-0.00176, 0.00428) | 0.508 | -0.01959 (-0.03065, -0.00243) | 0.032 | -5.5 (-58.8, 18.6) |

Note: SpO2 pre3: SpO2 change 3 hours after NO use (pre-ductal); SpO2 post3: SpO2 change 3 hours after NO use (post-ductal); Oi3: Oxygenation Index at 3 hours; Oi24: Oxygenation Index at 24 hours; Oi48: Oxygenation Index at 48 hours

**Table S4: Mediation analysis for the associations between Weight-Standardized Initial iNO dose and Moderate-to-Severe BPD**

| **Independent variable** | **Mediator** | **Total effect** | | **Indirect effect** | | **Direct effect** | | **Proportion mediated, % (95% CI)** |
| --- | --- | --- | --- | --- | --- | --- | --- | --- |
|  |  | **Coefficient (95% CI)** | **P value** | **Coefficient (95% CI)** | **P value** | **Coefficient (95% CI)** | **P value** |  |
| Initial iNO dose per body weight | SpO2_pre3 | -0.02814 (-0.04416, -0.01067) | <0.001 | 0.00012 (-0.00364, 0.00243) | 0.920 | -0.02826 (-0.04288, -0.01012) | <0.001 | -0.4 (-11.7, 13.4) |
| Initial iNO dose per body weight | **SpO2_post3** | -0.02871 (-0.04355, -0.01069) | <0.001 | -0.00105 (-0.00518, 0.00478) | 0.656 | -0.02765 (-0.04572, -0.01045) | <0.001 | 3.7 (-17.2, 19.5) |
| Initial iNO dose per body weight | oi3 | -0.02787 (-0.04361, -0.01062) | <0.001 | -0.00060 (-0.00491, 0.00138) | 0.564 | -0.02727 (-0.04200, -0.00980) | 0.004 | 2.2 (-6.4, 21.7) |
| Initial iNO dose per body weight | oi24 | -0.02817 (-0.04442, -0.01007) | <0.001 | 0.00015 (-0.00204, 0.00211) | 0.868 | -0.02832 (-0.04360, -0.01001) | <0.001 | -0.5 (-8.1, 7.5) |
| Initial iNO dose per body weight | oi48 | -0.02819 (-0.04397, -0.01008) | <0.001 | 0.00019 (-0.00231, 0.00314) | 0.984 | -0.02838 (-0.04469, -0.00947) | <0.001 | -0.7 (-14.1, 12.8) |

Note: SpO2 pre3: SpO2 change 3 hours after NO use (pre-ductal); SpO2 post3: SpO2 change 3 hours after NO use (post-ductal); Oi3: Oxygenation Index at 3 hours; Oi24: Oxygenation Index at 24 hours; Oi48: Oxygenation Index at 48 hours

| **Independent variable** | **Mediator** | **Total effect** | | **Indirect effect** | | **Direct effect** | | **Proportion mediated, % (95% CI)** |
| --- | --- | --- | --- | --- | --- | --- | --- | --- |
|  |  | **Coefficient (95% CI)** | **P value** | **Coefficient (95% CI)** | **P value** | **Coefficient (95% CI)** | **P value** |  |
| Maximum iNO dose per body weight | SpO2_pre3 | -0.01458 (-0.03147, 0.00225) | 0.116 | 0.00005 (-0.00368, 0.00284) | 0.928 | -0.01464 (-0.03256, 0.00341) | 0.112 | -0.4 (-41.4, 51.6) |
| Maximum iNO dose per body weight | **SpO2_post3** | -0.01498 (-0.03153, 0.00260) | 0.112 | -0.00072 (-0.00439, 0.00386) | 0.732 | -0.01427 (-0.03054, 0.00240) | 0.100 | 4.8 (-32.2, 60.6) |
| Maximum iNO dose per body weight | oi3 | -0.01434 (-0.03116, 0.00208) | 0.116 | -0.00115 (-0.00532, 0.00142) | 0.480 | -0.01320 (-0.02953, 0.00401) | 0.152 | 8.0 (-67.7, 76.5) |
| Maximum iNO dose per body weight | oi24 | -0.01457 (-0.03134, 0.00262) | 0.116 | 0.00004 (-0.00174, 0.00192) | 0.984 | -0.01461 (-0.03144, 0.00254) | 0.120 | -0.3 (-21.3, 25.5) |
| Maximum iNO dose per body weight | oi48 | -0.01459 (-0.03160, 0.00246) | 0.116 | 0.00078 (-0.00197, 0.00410) | 0.644 | -0.01536 (-0.03238, 0.00324) | 0.092 | -5.3 (-57.3, 54.5) |

**Table S5: Mediation analysis for the associations between Weight-Standardized Maximum iNO dose and Moderate-to-Severe BPD**

Note: SpO2 pre3: SpO2 change 3 hours after NO use (pre-ductal); SpO2 post3: SpO2 change 3 hours after NO use (post-ductal); Oi3: Oxygenation Index at 3 hours; Oi24: Oxygenation Index at 24 hours; Oi48: Oxygenation Index at 48 hours

**Table S6:Tests of Sphericity and Homogeneity of Variances**

|  | **Mauchly's W** | **p-value** | **Greenhouse-Geisser ε** | **Huynh-Feldt ε** |  |
| --- | --- | --- | --- | --- | --- |
| Times | 0.4262142 | <0.001 | 0.6617726 | 0.6796996 | Times |

**Table S7:Homogeneity of Variances Test (Levene's)**

|  | **F** | **df1** | **df2** | **p-value** |  |
| --- | --- | --- | --- | --- | --- |
| oi0 | 1.3412449 | 1 | 75 | 0.250 | oi0 |
| oi24 | 0.2582324 | 1 | 75 | 0.613 | oi24 |
| oi48 | 0.3609173 | 1 | 75 | 0.550 | oi48 |

*Note: Oi0: Worst oxygenation index before iNO treatment; Oi24: Oxygenation Index at 24 hours; Oi48: Oxygenation Index at 48 hours*
